# Appropriate provision of anti-D prophylaxis to RhD negative pregnant women: a scoping review

**DOI:** 10.1186/s12884-014-0411-1

**Published:** 2014-12-10

**Authors:** Trina M Fyfe, M Jane Ritchey, Christorina Taruc, Daniel Crompton, Brian Galliford, Rose Perrin

**Affiliations:** Northern Medical Program, University of Northern British Columbia, 3333 University Way, Prince George, BC V2N 4Z9 Canada; Northern Health - Corporate Office Suite 600, 299 Victoria St., V2L 5B8 Prince George, BC Canada

**Keywords:** RhD isoimmunization, practice guidelines, Rho(D) Immune Globulin, guideline adherence, anti-D immunoglobulin

## Abstract

**Background:**

The purpose of this scoping review was to review the literature on healthcare provider provision of anti-D prophylaxis to RhD negative pregnant women in appropriate clinical situations in various healthcare settings.

**Methods:**

A scoping review framework was used to structure the process. The following databases were searched: CINAHL (EBSCO), EBM Reviews (OvidSP), Embase (OvidSP), Medline (OvidSP), and Web of Science (ISI). In addition, hand searching of article references was conducted. The search yielded 301 articles. Thirty-five articles remained for review after screening. Two team members reviewed each article using a detailed data collection sheet. A third reviewer was utilized if discrepancies occurred amongst reviewers.

**Results:**

The review process yielded 18 included articles. The majority of the studies were conducted in the United Kingdom. Of the 18 studies, 15 were retrospective studies. The articles were largely conducted in one institution. The articles with a focus on routine antenatal provision of anti-D immunoglobulin found that it was given 80 to 90% of the time. Postpartum provision of anti-D immunoglobulin had significantly higher results of 95-100%. The review found that the delivery of anti-D immunoglobulin to RhD negative pregnant women during situations of potential sensitizing events was suboptimal.

**Conclusions:**

The included articles examine the management of RhD negative pregnancies in various countries with existing national guidelines. The existing evidence indicates an opportunity for quality improvement in situations where potential sensitizing events are not at routine times in pregnancy, such as miscarriage or fetal demise early in pregnancy. Routine care for the prevention of RhD alloimmunization in pregnancy and postpartum appears to be fairly consistent. The paucity of recent literature in this area leads to a recommendation for further research.

**Electronic supplementary material:**

The online version of this article (doi:10.1186/s12884-014-0411-1) contains supplementary material, which is available to authorized users.

## Background

RhD alloimmunization can lead to Hemolytic Disease of the Fetus and Newborn (HDFN) or in severe cases fetal demise [1]. This can occur if an RhD negative pregnant woman has a sensitizing event during her pregnancy that causes the development of anti-D antibodies [2]. These antibodies work to destroy fetal red blood cells [1].

The prophylaxis for the prevention of RhD alloimmunization was developed in the 1960s [1]. Since its discovery, anti-D immunoglobulin has remained the gold standard in the prevention of RhD alloimmunization and consequently HDFN [3]. However, this is under debate in the current literature [4]. Anti-D immunoglobulin is a blood product given to RhD negative pregnant women during pregnancy and after delivery. According to various guidelines, RhD negative women receive the prophylaxis at 28 weeks (and again at 34 weeks if the guideline indicates a two dose regime) and again after the delivery of an RhD positive fetus [5-10]. Outside of routine provision, RhD negative pregnant women can receive anti-D immunoglobulin during pregnancy when potential sensitizing events occur. A list of potential sensitizing events can be found in an additional file (see Additional file 1). Since the development of anti-D immunoglobulin, the rate of RhD alloimmunization and its consequences has been significantly reduced but the cited RhD alloimmunization rate remains at 6.7/1000 live births, which leaves room for improvement [11].

In 2012, two retrospective studies were published that examined the delivery of anti-D immunoglobulin to RhD negative pregnant women in appropriate clinical situations within emergency departments [12,13]. These two studies found that in certain clinical situations the delivery of anti-D immunoglobulin to RhD negative pregnant women was suboptimal. The recent emergence of these two studies led the authors of this review to question whether more research exists on the provision of prophylaxis to RhD negative pregnant women and if there are settings besides emergency departments that contribute to the RhD alloimmunization rate of 6.7/1000 births [11].

The purpose of this scoping review is to explore the literature on the provision of anti-D prophylaxis to RhD negative pregnant women in appropriate clinical situations in various healthcare settings. The hypothesis is that RhD negative pregnant women receive anti-D immunoglobulin in routine situations, such as within 72 hours after birth, but in situations of potential sensitizing events, such as miscarriage or fetal demise, anti-D immunoglobulin may not always be provided correctly or at all. To date, a knowledge synthesis has not been conducted on this topic.

## Methods

A scoping review framework was chosen because it is an exploratory process, enabling the team to determine the depth, range, and nature of the research that exists, thereby not limiting to specific types of research methodology and critical analysis. This review followed Levac et al’s [14] scoping review framework: identify the research question, identify relevant studies, select the studies, chart the data, summarize and report the results, and consult with knowledge users. Ethics was not required because this review methodology was not experimental research nor did it involve human participants.

### Data sources

The following databases were searched by a librarian: CINAHL (EBSCO), EBM Reviews (OvidSP), Embase (OvidSP), Medline (OvidSP), and Web of Science (ISI). In addition to database searching the librarian hand searched reference lists of potential articles to include. To ensure rigor, the librarian had the database search strategy peer reviewed by another librarian. This process reduced the likelihood of human based search error [15].

The following search terms were used: Rho(D) Immune Globulin, immunoglobulins, anti-idiotypic antibodies, anti-D immunoglobulin, anti-D immune globulin, anti-D prophylaxis, anti-D immunoprophylaxis, Rhogam, Winrho, pregnancy, pregnant women, Rh alloimmunization, Rh sensitization, Rh isoimmunization, Rh incompatibility, rhesus disease, blood group incompatibility, hospitalists, family physicians, emergency physicians, obstetricians, nurse practitioners, midwifery, nurse midwives, hospital emergency service, emergency department, acute care, obstetrics and gynecology department, primary care, outposts, ambulatory care facilities, hospital units, or birthing centres. Medical subject headings were used when available and deemed appropriate. Keyword searching utilized truncation and alternative spelling.

### Study selection

In order to explore the nature and size of the literature in this area the selection criteria for this synthesis were broad. After the search was complete, the results were combined and duplicates removed, the articles were screened for relevancy. After the screening process, two team members reviewed the full text of each article. A data extraction sheet was used to compile data on each article with the option of inclusion or exclusion. Reviewers were asked to solve discrepancies amongst themselves but in the event that they were unable to do this a third reviewer was brought in to resolve the discrepancy. A reviewer included an article if it addressed the provision of prophylaxis in routine and/or sensitizing situations within healthcare settings. Articles that explored dosage and/or the administration of anti-D immunoglobulin practices were excluded.

### Potential sensitizing events

The authors define a sensitizing event in RhD negative pregnant women as an event that leads to the development of anti-D antibodies due to maternal-fetal blood exchange. The following list of potential sensitizing events was adapted from Urbaniak & Greiss’ [2] and incorporated events listed in existing guidelines. The guidelines consulted in this process were from the World Health Organization, American Congress of Obstetricians and Gynecologists in the United States (US), the National Institute for Clinical Excellence in the United Kingdom (UK), Australia’s National Blood Institute, the Society of Obstetricians and Gynecologists of Canada, and the British Committee for Standards in Haematology [5-9,16]. An additional file provides a list that summarizes all clinical events considered at risk for sensitization in RhD negative pregnant women because of the potential for fetal-maternal hemorrhage as outlined in the aforementioned guidelines (see Additional file 1) [1].

### Summarizing and reporting results

Each reviewer completed a data extraction form for each article reviewed. The form included four predefined themes: policy, practice, education, and research. The reviewers were asked to provide comments regarding the articles’ contribution, challenges, or opportunities for each of the themes. A narrative discussion was used to synthesize the studies according to the four predefined themes. The articles were categorized and summarized into a matrix: methodology, geographic location, healthcare setting, number of participants, and if the study looked at the provision of anti-D immunoglobulin for routine antenatal, routine postnatal, and/or sensitizing events. An additional file provides the matrix of included articles (See Additional file 2).

## Results

The searches yielded 323 articles. After duplicates were removed 301 articles remained. The screening process weeded out 266 abstracts, leaving 35 articles for review. Hand searching references of the 35 articles identified another 13 articles for potential inclusion in the review. The final reviewing process included a total number of 18 articles. The review process is illustrated in an additional file using a flow chart (see Additional file 3).

Of the 18 articles included in the review, 13 were conducted in the UK, two in Canada, one in Australia, one in the US, and one review article. The settings included maternity units, nurse led clinics, emergency departments, and general practice clinics.

The majority, 15 articles, of the included studies are retrospective cohort studies. There was only one prospective cohort study available. Of the retrospective studies 5 involved more than one institution, 7 articles were at one institution, and two articles were not clear on the setting. Of the 15 retrospective studies one utilized data from a national reporting system.

### Routine antenatal

According to the guidelines in the UK, Australia, Canada, and the US, anti-D immunoglobulin should be given routinely to RhD negative pregnant women [5,7-10]. The guidelines state that the prophylaxis may be given in two injections at 28 and 34 weeks or in one injection at 28 weeks. Of the 18 articles, 8 explored adherence to the delivery of routine antenatal anti-D immunoglobulin [12,17-23]. Each article varied in the approach but the overall findings are that the delivery of anti-D immunoglobulin in these routine situations is consistently delivered. The figures ranged from 80 to 90% adherence. MacKenzie et al’s [20] study found the timing of the delivery of anti-D prophylaxis was an issue: “There was limited success at providing prophylaxis at the correct gestation for eligible women. Eighty-nine percent received one injection and 74% received both, but only 29% at the correct gestation.” In a 2012 Canadian study, Koby et al. [12] found that the routine postnatal delivery of anti-D immunoglobulin occurred 98.5% of the time, whereas the antenatal delivery of the prophylaxis was suboptimal at 85.7%. The authors suggest that hospital-based protocol systems lead to increased adherence in postnatal administration [12]. The hospital-based protocol described involves various checkpoints throughout labour and delivery. These checkpoints alert physicians and nurses to note and deliver anti-D immunoglobulin (if needed) at admission, post-delivery, transfer to postpartum unit, and at discharge [12]. The authors hypothesize that the administration rate of antenatal anti-D immunoglobulin is lower due to errors that may occur in physician-dependent situations, such as failure to identify and treat RhD negative women and lack of protocols and/or team based approaches [12].

### Routine postnatal

The provision of anti-D immunoglobulin to RhD negative women upon the delivery of an RhD positive infant should occur within 72 hours [1]. The studies included in this review found favourable results in this regard. The routine provision of postnatal anti-D immunoglobulin was fairly consistent across the studies. Studies found that RhD negative women that delivered an RhD positive infant were given anti-D immunoglobulin between 95-100% of the time [12,19,24,25].

### Sensitizing events

As defined earlier, potential sensitizing events can occur at any time throughout the pregnancy and can have devastating consequences. Out of the 18 articles included in this review 8 articles examined the provision of anti-D immunoglobulin when potential sensitizing events occur [13,23,24,26-30].

The testing of RhD status is an integral step in the prevention of RhD alloimmunization. In situations for which potential sensitizing events present themselves pregnant women were often discharged without having their RhD status tested. Further, pregnant women that were discharged may have been RhD negative consequently raising the opportunity for RhD alloimmunization to occur. A retrospective study conducted in Canada in 1990 (prior to national guidelines), found that the RhD status of pregnant women discharged from the emergency department was performed in 86% of all the women discharged [27]. A more recent study conducted in the US found that 89% of women with potential sensitizing events had their RhD status documented and/or tested [13]. A small study exploring the effectiveness of a nurse practitioner led early pregnancy clinic found that all RhD negative women presenting to the clinic had their RhD status tested and received appropriate prophylaxis [26].

This review found that the provision of anti-D immunoglobulin was low in situations for which potential sensitizing events occur. A recent US study found that although 89% had RhD status testing, only 54% of those that were RhD negative actually received the prophylaxis and was lower in second and third trimesters [13]. In an older study conducted in the UK, adherence was looked at prior to 12 weeks and after 12 weeks gestation in women experiencing potential sensitizing events based on available guidelines at the time; in each instance the provision of anti-D immunoglobulin was suboptimal [24]. For example, in the second and third trimester “more than 25% of women in the large maternity unit, and over 33% in the smaller unit” did not receive anti-D immunoglobulin [24].

Gestational age appears to be a factor in the suboptimal provision of anti-D immunoglobulin. A retrospective study looking at the management of women presenting the emergency department prior to 12 weeks gestation found that of 112 patients 97 were discharged without having their RhD status tested [30]. A survey study conducted in Australia found that general practitioners would offer anti-D immunoglobulin in only 57% of cases of threatened miscarriages [29]. The general practitioners were also more likely to provide anti-D immunoglobulin if the patient presented with heavy bleeding or if the pregnancy was non-viable [29]. Interestingly, this study found that in cases of threatened miscarriage, “rural doctors were more likely than urban doctors to offer anti-D in this situation (66% vs. 55%; difference, 11%; 95% CI, 1% to 22%)” [29]. An earlier study in the UK “found a significant level of noncompliance with published recommendations in relation to routine screening for antibodies, administration of anti-D immunoglobulin and Kleihauer testing” [23]. Although this study is older and guidelines have since changed, the findings suggest there was an issue with the provision of anti-D immunoglobulin less than 20 weeks gestation [23].

## Discussion

The literature provides evidence that there are opportunities for quality improvement in the delivery of prophylaxis in routine and clinically significant situations. RhD negative women are not being consistently tested for their RhD status in clinically significant situations and are consequently not provided anti-D immunoglobulin when required. The categories of practice, policy, education, and research were chosen to discuss the results as it pertains to each category. The attempt is to provide suggestions and guidance in each domain.

### Practice

There is a need for an increased efficiency with the provision of anti-D immunoglobulin, particularly in situations of potential sensitizing events. The existing research provides the evidence that anti-D is not always given at the right time or at all; although, in controlled environments such as maternity wards RhD negative women are receiving prophylaxis post-delivery almost 100% of the time.

Only two studies discussed the woman’s role in the decision to receive anti-D immunoglobulin [18,21]. In some instances the women made well-informed decisions based on their current health and relationship status. In MacKenzie et al’s [21] study, they found a small number of women increasingly denying prophylaxis in the 1990s. The authors entertained the notion that this rise in refusal of anti-D was attributed to “a growing anxiety about possible infection from the administration of blood products during the decade, and such anxiety may well have been exacerbated when the preparation previously used for RhD prophylaxis was withdrawn because of concerns relating to variant Creutzfeldt–Jakob disease transmission” [21]. These results suggest that there is a need for improved communication amongst health care providers and RhD negative women, between departments (such as laboratories and emergency departments), and between health care providers involved in the continuum of care.

The studies included in this review span twenty-two years, the earliest study dating back to 1992. The last ten years has seen only six studies addressing this topic despite recent guideline development and implementation [12,13,17,18,21,31]. These five studies (and one review) continue to find opportunities for improved management of RhD negative pregnancies, particularly in situations for which sensitizing events occur. Guidelines on the prevention of RhD alloimmunization do not provide strong recommendations for situations involving sensitizing events, particularly in the first trimester. This is a result of a paucity of evidence regarding the effectiveness of anti-D immunoglobulin in the first trimester after a sensitizing event [16,32]. This lack of evidence is one of the reasons guidelines are lacking strong recommendations. Consequently, the delivery of anti-D immunoglobulin continues to be problematic and quality improvement remains suboptimal.

Few studies provide recommendations for an improvement in the delivery of anti-D immunoglobulin. The most recent Canadian study suggests that improved communication and patient education for RhD negative pregnant women would potentially improve adherence. The same study suggests that a checklist system, such as the one described in the results section discussing post-natal administration of anti-D immunoglobulin would be helpful in antenatal situations. A team-based approach involving nurses with specific checklists in place and/or a clinic specific to the administration of anti-D immunoglobulin are other recommendations put forth by Koby et al. [12]. These suggestions need to be integrated into the management of RhD negative pregnancies both in hospital and primary care settings. These interventions need to be evaluated for effectiveness and quality improvement.

### Policy

The longest retrospective study of 15 years conducted in the UK, provides data that there has been consistent errors of omission or late delivery of anti-D immunoglobulin [17]. This study is important because it covers a lengthy period of time in reporting of anti-D immunoglobulin mismanagement but it also covers the period of guideline implementation. Throughout the 15 years of reporting new guidelines were disseminated. Despite the new guidelines mismanagement continued to occur. Perhaps continued issues are due to an increase in healthcare providers reporting or a lack of uptake or clarity of the guidelines, but nevertheless an issue regarding the delivery of anti-D immunoglobulin still occurred. Inevitably there is room for improvement in the delivery of anti-D immunoglobulin and the need for clearer guidelines with implementation plans and evaluation to ensure the uptake of evidence.

In several studies conducted in acute care settings, such as emergency departments, pregnant women with potential sensitizing events did not receive optimal care. This suggests that there are opportunities for this clinical setting to develop interventions that perhaps integrate RhD testing and the increased delivery of anti-D in clinically significant potential sensitizing situations. The opportunity for quality improvement in the management of RhD negative pregnancy is imperative.

### Education

Thorpe’s review article is an example of an educational attempt to improve the quality of care for RhD negative pregnant women with a focus on blunt trauma [31]. In Nova Scotia there is an active continuing medical education program and reporting system for RhD negative pregnancies [33]. These two examples provide strategies for continued education in this area. Based on the results of this review continued education should focus on routine antenatal and potential sensitizing situations. In addition, continued education regarding communication would help to improve the quality of care by ensuring the issues related to mismanagement are not caused by communication factors. An Australian study found that rural physicians were more likely to deliver anti-D immunoglobulin in situations of threatened miscarriages than urban physicians [29]. Although the definition of rural is not provided and the study is a self-reported survey of individual practices, an exploration into what aspects of rural practice and/or education that lead to the increased provision of anti-D in situations of threatened miscarriage by rural physicians would be helpful.

The definition of shared-care states that patients are provided with the opportunity to engage and collaborate in health care decision-making [34]. Only two studies mentioned the role of RhD negative women in the management of their pregnancy [18,21]. The limited literature in this area requires further exploration. Shared care and patient engagement literature provides evidence that patients require knowledge and information in order to engage in their care. Therefore, it is suggested that women need to be informed of their blood type, perhaps prior to pregnancy, and educated about RhD factor and the risks that lie therein.

### Research

The retrospective cohort methodology is an appropriate method in researching the use of anti-D immunoglobulin in clinical settings [35]. It would be helpful to have more studies utilizing population-based data, large multi-center studies involving prospective approaches or retrospective approaches, and more studies in Canada, Australia, and the US. Further research is required to understand the factors associated with suboptimal provision of anti-D immunoglobulin in situations where potential sensitizing events occur. As Koby et al. [12] suggest, errors of omission can occur in situations where the decisions are physician-dependent. However, only one study provided the factors involved in the omission of or late administration of anti-D immunoglobulin [17]. The factors involved poor documentation, misinterpretation of laboratory results, issues with storage of the prophylaxis, and communication between departments. In addition, a better understanding of women’s knowledge and experiences with RhD negative pregnancies and its possible implications would provide further insight into RhD alloimmunization. Once there is a basic understanding interventions may be developed and trialed for effectiveness.

### Limitations

The limitations of this scoping review lie within the literature retrieved and included. The studies included span across several decades with the first article published in 1992 [27]. In addition, these included studies have been conducted in four different countries. The majority of articles were conducted in the UK. This means that the results of these studies are not necessarily generalizable across various countries, amongst varying clinical guidelines, with current clinical guidelines, and within different settings. Due to these limitations conducting a further systematic review of the literature is not recommended.

## Conclusions

The included articles offer a glimpse into the management of RhD negative pregnancies in various countries with existing national guidelines. The existing evidence indicates an opportunity for quality improvement in situations where potential sensitizing events are not at routine times in pregnancy, such as miscarriage or fetal demise early in pregnancy. Routine care for the prevention of RhD alloimmunization in pregnancy and postpartum appears to be fairly consistent. The paucity of recent literature in this area leads to a recommendation for further research.

## Additional files

Additional file 1: Figure S1. Potential sensitizing events. Additional file 2: Table S1. Matrix of included articles. Additional file 3: Figure S2. Flowchart of review process.

## Abbreviations

APH: Antepartum haemorrhage
CVS: Chorionic villus sampling
FBS: Fetal blood sampling
HDFN: Hemolytic Disease of the Fetus and Newborn
IUD: Intrauterine death
UK: United Kingdom
US: United States
