# Supplementary material for: Appropriate provision of anti-D prophylaxis to RhD negative pregnant women: a scoping review
Source: BMC Pregnancy Childbirth. 2014 Dec 10;14:411. doi: 10.1186/s12884-014-0411-1 (PMC4265333; doi:10.1186/s12884-014-0411-1)
Supplement: Additional file 1: Figure S1. — Potential sensitizing events. [file 12884_2014_411_MOESM1_ESM.docx]

**Additional file 1: Figure S1 – Potential sensitizing events**

- Delivery of an RhD positive infant (or if the infant’s Rh type is unknown)
- Abortion
  - Therapeutic termination of pregnancy
  - Spontaneous abortion followed by instrumentation
  - Spontaneous complete or incomplete abortion after 12 weeks gestation
  - Threatened abortion before 12 weeks (when bleeding is heavy or repeated or is associated with abdominal pain)
  - Threatened abortion after 12 weeks (when bleeding continues intermittently after 12 weeks gestation)
- Invasive prenatal diagnosis
  - Amniocentesis
  - Chorionic villus sampling (CVS)
  - Cordocentesis
  - Fetal blood sampling (FBS)
- Other intrauterine procedures
  - Insertion of shunts
  - Embryo reduction
  - Transfusions
  - Surgery
  - Laser
- Intra-operative cell salvage
- Antepartum haemorrhage (APH)/Uterine (PV) bleeding in pregnancy
- External cephalic version
- Abdominal trauma
- Ectopic pregnancy
- Molar pregnancy
- Intrauterine death (IUD)
- Stillbirth
