# Supplementary material for: Appropriate provision of anti-D prophylaxis to RhD negative pregnant women: a scoping review
Source: BMC Pregnancy Childbirth. 2014 Dec 10;14:411. doi: 10.1186/s12884-014-0411-1 (PMC4265333; doi:10.1186/s12884-014-0411-1)
Supplement: Additional file 2: Table S1. — Matrix of included articles. [file 12884_2014_411_MOESM2_ESM.docx]

**Additional file 2: Table S1 – Matrix of included articles**

| **Author(s) & Year** | **Geographic location** | **Methodology** | **Setting** | **Number of participants (dates)** | **Issue** |
| --- | --- | --- | --- | --- | --- |
| Bolton-Maggs et al, 2013 | UK | Retrospective cohort study | Serious Hazards of Transfusion reporting system | 1211  (5 years, 1996-2011) | Routine antenatal |
| Chaffe et al, 2007 | UK | Retrospective cohort study | 2 hospitals | 207  (2004, 2 different time periods) | Routine antenatal |
| Fox et al, 1999 | UK | Retrospective cohort study | 1 early pregnancy assessment clinic | 200  (6 months) | Potential Sensitizing event(s) |
| Ghosh & Murphy, 1994 | UK | Retrospective cohort study | 2 regions | Region A = 296  Region B = 824  (8 months, 1992) | Routine postnatal  Potential  Sensitizing event(s) |
| Grant & Hyslop, 1992 | Canada | Retrospective cohort study | 1 hospital emergency department | 138  (3 months, 1990) | Potential  Sensitizing event(s) |
| Griffey et al, 2012 | US | Retrospective cohort study | 1 hospital emergency department | 808  (1 year, 2009-2010) | Potential  Sensitizing event(s) |
| Howard et al, 1997 | UK | Retrospective cohort study | 7 maternity units | 922  (8 months, 1994) | Routine postnatal  Potential  Sensitizing event(s) |
| Hughes et al, 1994 | UK | Retrospective cohort study | 1 region (not clear number of hospitals included) | 80  (5 years, 1985-1990) | Routine antenatal  Routine postnatal  Potential  Sensitizing event(s) |
| Huggon & Watson, 1993 | UK | Retrospective cohort study | 1 hospital emergency department | 39  (1 month, 1992) | Potential  Sensitizing event(s) |
| Koby et al, 2012 | Canada | Retrospective cohort study | 1 hospital | 1868  (5 years, 2001-2006) | Routine antenatal  Routine postnatal |
| MacKenzie et al, 1999 | UK | Retrospective & prospective cohort study | 3 health districts (2 counties) | Retrospective  County A = 2931  County B = 3040  (6 years, 1980-1986)  Prospective  County A = 3320  County B = 3146  (6 years, 1990-1996) | Routine antenatal |
| MacKenzie et al, 2006 | UK | Retrospective cohort study | 1 hospital | 365  (1992-1996)  215  (1997-2003) | Routine antenatal |
| Mayne et al, 1997 | UK | Retrospective cohort study | 1 city | 5000  (7 years, 1988-1995) | Routine antenatal |
| McLaren & Shelley, 2002 | Australia | Survey | 1 state | 382 physicians  (4 months, 1997-1998) | Potential  Sensitizing event(s) |
| McSweeney et al, 1998 | UK | Retrospective cohort study | 15 obstetrics units | 147  (3 years, 1988-1991) | Routine antenatal |
| Rennie et al, 2001 | UK | Retrospective cohort study | 1 hospital | 288  (8 months, 1999-2000) | Potential  Sensitizing event(s) |
| Thorp, 2008 |  | Review article |  |  | Potential  Sensitizing event(s) |
| Weinberg, 2001 | UK | Retrospective cohort study | 1 hospital | 112  (17 months, 1998-1999) | Potential  Sensitizing event(s) |
