# Supplementary material for: Appropriate provision of anti-D prophylaxis to RhD negative pregnant women: a scoping review
Source: BMC Pregnancy Childbirth. 2014 Dec 10;14:411. doi: 10.1186/s12884-014-0411-1 (PMC4265333; doi:10.1186/s12884-014-0411-1)
Supplement: Additional file 3: Figure S2. — Flowchart of review process. [file 12884_2014_411_MOESM3_ESM.docx]

**Additional file 3: Figure S2 - Flowchart of review process**

Hand search

N = 13

Duplicates removed

N = 22

**Total articles after review N = 18**

Hand search = 3

Database search = 15

Removed after screening

N = 266

Full text remaining after screening process

N = 35

CINAHL (Ebsco)

EBM Reviews (OvidSP)

Embase (OvidSP)

Medline (OvidSP)

Web of Science (ISI)

N = 323

After duplicates removed

N = 301
